# Weaner production with low antimicrobial usage: a descriptive study

**DOI:** 10.1186/s13028-015-0130-2

**Published:** 2015-07-17

**Authors:** Mette Fertner, Anette Boklund, Nana Dupont, Claes Enøe, Helle Stege, Nils Toft

**Affiliations:** Section for Epidemiology, National Veterinary Institute, Technical University of Denmark, Bülowsvej 27, 1870 Frederiksberg C, Denmark; Department of Large Animal Sciences, Faculty of Health and Medical Sciences, University of Copenhagen, Grønnegaardsvej 2, 1870 Frederiksberg C, Denmark

**Keywords:** Swine, pig, Antibiotic use, Management, Health

## Abstract

**Background:**

Health, productivity and antimicrobial use in the production of pigs are expected to be interrelated to some extent. Previous studies on register-based data have investigated these correlations with a subsequent large variation residing at the farm level. In order to study such farm factors in more detail we designed an elaborate interview-guide. By in-depth interviews of farmers with well-managed 7–30 kg (weaner) productions we sought to describe a set of common key-factors characterizing their management practices. Identification of such common practices could be used in follow-up projects, investigating whether identified factors really are characteristic for good-practicing famers.

**Results:**

Eleven farms were selected for a farm visit and in-depth interview. Participating farms used less antimicrobials than the national median (8.2 animal daily doses/100 weaners/day), had a mortality below the national average (2.9%) and an average daily weight gain above the national average (443 g/day). Similarities were observed among participating farms, including the sectioning of farms, use of all-in-all-out procedures with subsequent cleaning, purchasing 7 kg weaners from only one source, as well as active participation in management by a committed farm owner. Most farmers had a specific point of focus in their management, and were convinced that this was the reason for their success. This included; feeding, treatment strategy, refurbishment of facilities and presence in the shed.

**Conclusion:**

According to register data, participating farms were alike; in the good league regarding use of antimicrobials, mortality and daily growth. However, on-farm interviews elucidated more heterogeneity among farmers than expected. Most of the farmers had a specific point of focus, which they considered to be crucial for their good results. These results indicate the importance of non-registerable factors, highlighting the value of qualitative study techniques in the understanding of human actions. Further studies on the effect of various farmer types are recommended.

## Background

Several databases with information regarding farm characteristics, infection status and antimicrobial use in pig farms are available in Denmark. Previous studies have investigated how much of the between-farm variation in antimicrobial use can be attributed to risk factors present in such registers. Variation at farm level has been found to constitute 38% [1] and 40% [2] of the total variation, underlining the importance that management, housing and the individual farmer have on the use of antimicrobials. Alternative study designs are therefore required to augment the value of register-based data.

Health, productivity and antimicrobial use at a farm are expected to be interrelated to some extent. Growth-enhancing effects of antimicrobials added to the feed in sub-therapeutic concentrations are well-known [3]. Furthermore, studies on the effect of phasing out growth promoters have shown an increased incidence of gastrointestinal disorders among weaners in Denmark [4]. Sweden experienced an increased post-weaning mortality and decreased growth rate among weaners [5], which was not confirmed in Denmark [6].

Antimicrobials prescribed for animals are reserved for therapeutic and metaphylactic purposes in the European Union [7]. A clear link can therefore be expected between the incidence of disease and amount of antimicrobials used. Additionally, it is recognized that a number of management-related parameters, as well as variation in treatment procedures can influence the use of antimicrobials in terms of disease-preventing initiatives. Procedures for disease prevention such as sectioning [8], hygiene [8] and handling of diseased pigs [9] have been negatively correlated with the use of antimicrobials. Due to its close link with gastrointestinal disorders [10, 11], feeding is also expected to have a significant influence on the use of antimicrobials in weaners. Treatment-related factors include the farmers’ perception of metaphylaxis, the ability to identify clinically-diseased pigs, and compliance with veterinary recommendations for treatment.

Denmark produces more than 30 million fattening pigs per year and is one of the world’s largest exporters of pork [12]. Among the European countries producing a similar amount of pork (Germany, Spain, France, Poland, Italy and the Netherlands) [12], Denmark has the lowest rate of antimicrobial use per animal [13]. Due to the large number of animals involved, pig production accounted for 76% of the veterinary antimicrobials prescribed in Denmark in 2012 [14] and it therefore receives the main political focus in terms of antimicrobial use. Calculated as animal daily doses (ADD) the majority of prescribed antimicrobials for pigs are administered for weaners (7–30 kg pigs), and mainly for gastrointestinal disorders [15, 16]. Since 2000, the amount of prescribed antimicrobials for all farms has been recorded in a Danish national database, VetStat [17]. However, parameters such as health, productivity and management practices for 7–30 kg pigs are not available in any national register. Average daily weight gain and mortality may be used as objective proxies for health and productivity, since diseased pigs are expected to have a reduced weight gain and may die [18]. Yet both these parameters are solely recorded on-farm, complicating the access of these data.

Our study examines well-managed farms: farms, which have overcome the apparent paradox of having a low rate antimicrobial use, simultaneously combined with low mortality and high productivity. It was our hypothesis that well-managed 7–30 kg (weaner) productions have a set of common key-factors characterizing their management practices. Using a semi-qualitative study design, we were able to obtain a detailed knowledge about the farms and their owners. This allowed us to further elucidate issues on which it is not possible to make inferences based on the information from national databases. The objective was to identify management-related factors which, according to the farmers’ own perceptions, were the primary reasons for their positive results.

## Methods

Participating farms were identified by the following selection procedure. Eleven veterinarians working in pig practice and representing different geographical regions and various veterinary practices, were contacted by telephone. Of these, seven agreed to participate in the study. They were encouraged to send a list of their clients with the lowest rates of antimicrobial use, and the highest rates of health and productivity. To fulfill the selection criteria, farms had to produce 7–30 kg pigs and not be organic or free-range. Each veterinarian selected three to eight of their affiliated pig farms, giving a total of 46 farms. The amount of prescribed antimicrobials for each of the farms was subsequently calculated, based on VetStat data. The national database VetStat receives information on prescribed antimicrobials from feed mills, veterinarians and pharmacies [15, 17]. Data reported by pharmacies (comprising more than 98% of all antimicrobials prescribed for pigs) for the period of January 1 to December 31, 2012, were included in this study. Antimicrobials were quantified as ADD [19, 20]. The number of ADD prescribed for weaners was aggregated for each farm and divided by the number of weaner days multiplied by 100. This standardized unit (ADD/100 weaners/day) approximates the daily percentage of weaners treated at the farm. Information on the number of weaners present in each farm was extracted from the Central Husbandry Register (CHR). The number of weaners at the farm was multiplied by 366 days (number of days in 2012) in order to compute the total number of weaner days.

Of the 46 farms initially selected, only those using less antimicrobials than the median of all Danish farms (8.2 ADD/100 weaners/day) were considered further. These 32 farms were contacted by telephone. If the farmers were interested in participating, they were required to forward their efficiency control. Efficiency control is a voluntary registration, which some farmers use to keep track of productivity. From the efficiency control, mortality and average daily weight gain were used as objective proxies for health and productivity at the farm. Only the farms with weaners with an average daily weight gain above the Danish average (443 g/day for weaners (7–30 kg)) and a mortality below the Danish average^a^ (2.9%) were included in the study [21]. Farms with new infections were excluded, due to the risk of fluctuating management practices.

Eleven farms that fulfilled the inclusion criteria agreed to participate. These farms were visited and the person in charge of the production was interviewed by the corresponding author. Whenever possible, farm visits were carried out alongside the monthly veterinary advisory service visit. All visits were executed during February and March 2013. Each farm visit lasted between 2 and 4 h.

The interviews were structured in a semi open-ended manner, as described by Kvale and Brinkmann [22]. The structure of the interview was further discussed with an experienced interviewer. Due to the delicate topic of discussion, the decision was made not to record the conversations. The interview guide is available upon request. Typically, a farm visit started with a general assessment of the farm, conducted in association with the veterinarian. A thorough explorative interview was then conducted. Parameters expected to influence the antimicrobial use, health, and productivity at the farm including; employees, housing, management, hygiene, feed, biosecurity, movement of pigs and treatment procedures were addressed in the interview. Identification of these eight categories of questions were based on literature review prior to the study and subsequently presented to two specialized pig veterinarians to ensure inclusion of all important risk factors. Additionally, the farmer was asked what he/she considered the primary reasons for their successful production results. Five veterinarians were affiliated with these eleven farms and were interviewed separately. Veterinarians were first asked what they saw as the most important factors for a successful weaner production, and then they were asked to characterize their participating farms.

## Results and discussion

The results presented in Table 1 represent factors which were mentioned by farmers and veterinarians as possible key-factors: SPF^b^ infection status, management, internal biosecurity, pen hygiene between batches, feeding and treatment procedures.

**Table 1:** Characteristics of 11 Danish weaner producing farms with low use of antimicrobials and high productivity

	1^a^ (A and B^b^)	2 (A and B)	3	4	5	6
Farm demographics						
Infection status	Unknown	+Myc^c^	+Ap(6 + 12), +Myc, +PRRS	Free of all SPF pathogens	+PRRS	Unknown
Biosecurity	Non-SPF	SPF	SPF	SPF, closed farm^d^	SPF, closed farm	Non-SPF
Number of 7–30 kg pigs	3,000 + 3,000	2,400 + 1,800	4000	1,250	2,000	1,800
Supplier	Regular sow farm	Own sow farm	Own sow farm	Own sow farm	Own sow farm	Regular sow farm
Housed days^e^	~140	~52	45	57	52	139
Weight (kg), entrance– exit	7–slaughter	6.5–32	8.5–28	6.6–33	7.6–30	7.8–slaughter
AM^f^ usage (ADD_15_/100/day)	4.16 and 7.19	3.9 and 5.7	2.7	7.37	6.03	0.6
Average daily weight gain (g/day)	800–825	~500	462	464	~500	705
Mortality (%)	~2	~2.5	1.5	1.2	0.7	1
Management						
Staff experience (years)	2	5	3	10 + (owner)	10+	10+ (owner)
Owner participating	With feeding	Daily	At delivery	Daily	No	Daily
Hours spent/week^g^	20 (3.3 h/1,000w)	7 (1.7 h/1,000w)	37 (9.3 h/1,000w)	11 (8.8 h/1000w)	15 (7.5 h/1000w)	NA
Sorting by	Size and sex	Size	Size and sex	Size	Size	Size and sex
Sorting frequency	Continuously	Twice	Twice	Twice	At entrance	Once
Internal biosecurity						
Sectioning	High	Not 100%^h^	High	High	High (for 80%)	Not 100%
Vaccinate weaners^i^	PCV2	PCV2	No	No	PCV2	NA
Pen hygiene between batches						
Beyond washing	Disinfection	–	Disinfection	–	–	Disinfection
Drying (days)	2	3–10	6	2–6	3–5	7 days
Incl. heat (days)	2	2–3	2	1	1–3	1–2
Feeding						
Type	Home-mixed wet + lactic acid bacteria	Home-mixed dry	Purchased pelleted	Home-mixed dry	Home-mixed wet	Home-mixed dry
No. of mixtures	3 (7–9 variations)	2+ extra	2	2	3 + extra	2
Zinc first 2 weeks	No	Yes	Yes	Yes	Yes	Yes
Treatments						
Primary indication	Unthrifty	Diarrhea at shift in feed	Diarrhea at shift in feed	Diarrhea 3 weeks after weaning	(Diarrhea for the 20% not-sectioned)	Unthrifty
Method	Injection or AM in feed in sick pen	Group (section)	Injection only	Group (pen)	Group (water in feed trough)	Injection
% treated per batch^j^	5%	50%	NA^k^	NA	20%	NA

	7	8	9	10	11	
Farm demographics						
Infection status	+Myc	Free of all SPF pathogens	Free of all SPF pathogens	+Myc, +Ap6, +Ap12	+Myc, +PRRS	
Biosecurity	SPF	SPF	SPF	SPF	SPF	
Number of 7–30 kg pigs	2,200	4,000	4,000	3,300	1,720	
Supplier	Own sow farm	Own sow farm	Own sow farm	Own sow farm	Regular sow farm	
Housed days	44	50	56	55	55	
Weight (kg), entrance–exit	8.1–32	6.6–31.7	7.2–34.8	7.0–33.5	6.7–30.1	
AM usage (ADD_15_/100/day)	3.64	7.36	6.82	2.99	6.57	
Average daily weight gain (g/day)	576	497	498	486	426^l^	
Mortality (%)	1.6	0.8	1.4	1.7	1.6	
Management						
Staff experience (years)	6	1	5+	1	10+ (owner)	
Owner participating	Yes	Yes	Yes	Yes	Daily	
Hours spent/week	7 (3.2 h/1,000w)	37 (9.3 h/1,000w)	NA	14 (4.2 h/1,000w)	10 (5.8 h/1,000w)	
Sorting by	Size	Size	Size	Size	Size	
Sorting frequency	Twice	Continuously	Once	Once	Once	
Internal biosecurity						
Sectioning	High	Not 100%	Not 100%	High	Not 100%	
Vaccine weaners	No	No	No	No	NA	
Pen hygiene between batches						
Beyond washing	Disinfection	Disinfection	Disinfection	Disinfection	–	
Drying (days)	13	4	7-10	1	2	
Heating (days)	3	4	NA	1	2	
Feeding						
Type	(1) Purchased pelleted (2) Homemixed wet	Purchased pelleted	Purchased pelleted	Purchased pelleted	Purchased pelleted	
No. of mixtures	2	3+ extra	3	3	3	
Zinc first 2 weeks	Yes	Yes	Yes	Yes	Yes	
Treatments						
Primary indication	Diarrhea at shift in feed	Diarrhea at shift in feed	Diarrhea at shift in feed	Diarrhea 4–5 weeks after weaning	Diarrhea at shift in feed	
Method	Group (half section)	Group (section)	Group (section)	Group (section)	Group (section)	
% treated per batch	30–40%	100%	100%	100%	100%	

The information presented in the table is obtained through registrations from VetStat, the farmers efficiency controls as well as semi-qualitative on-farm interviews.

^a^Farm No 1 and No 2 did not present their efficiency control, but reported estimated results on mortality and daily weight gain.

^b^A and B indicates that the farmer has two herds with 7–30 kg pigs.

^c^Presence of SPF pathogens, see endnote description.

^d^Closed SPF farms produce their gilts themselves and therefore do not receive pigs from other farms.

^e^The average number of days that a batch of weaners remains in a section.

^f^ *AM* Antimicrobial.

^g^Labor hours spent per week is the number of weekly hours spent per 1,000 weaners, estimated by the farmer.

^h^“Not 100%” indicates defects in the sectioning procedures, such as: Weaners entering/leaving the housing having to pass through other sections, or pigs falling behind their batch mates being moved to another section.

^i^Informed by the herd owner, with the exception of farms 7, 8 and 9, where prescribed vaccines for weaners were obtained from VetStat.

^j^The percentage of pigs per batch being treated at least once during the weaner period, estimated by the farmer. Group treatment (“Group”) was administered through the drinking water if nothing else is stated.

^k^Not available.

^l^Farm 11 was included, despite an average daily weight gain below 443 g/day, due to an entrance weight (6.7 kg) considerably lower than the national average (7.2 kg).

In general, there was wide variation amongst farmers regarding their perception of which management parameters were the reasons for success in terms of low mortality, high daily weight gain and limited use of antimicrobials. They seemed to be divided into various categories with different points of focus, including feeding, presence in the shed, investment in facilities and treatment strategy. The choice of strategy seemed to be highly individual to each farmer. A committed farm owner, identified as a solid interest and participation in the management, characterized all participating farms. There were common factors among the interviewed farms, for example each received their 7 kg weaners from a single supplier, they implemented a high degree of sectioning, and a more or less consistent all-in-all-out production with cleaning between batches.

### Farm demographics

All farms received 7 kg weaners from one single supplier; either their own or a regular sow farm. The weight at entrance varied from 6.5 kg to 8.5 kg. Some farmers prioritized heavy weaners at entrance (Farms 3 and 7). Nine of the participating farms participated in the voluntary SPF program, insuring that 7 kg weaners also originate from a SPF sow farm. The quality of 7 kg weaners, in terms of e.g. weight, health and growth potential is expected to be interrelated with the management at the sow farm. However, it was out of the scope for this project to go into further detail regarding management in the sow farm.

Three farms were free of all SPF-registered pathogens (Farms 4, 8 and 9), while other six SPF farms had a varying number of registered pathogens (Farms 2, 3, 5, 7, 10 and 11). Typically, the farms took into account their infection status in the management practices, in terms of sectioning and vaccine programs. SPF-registered pathogens were commonly screened, while surveillance of gastrointestinal disorders was uncommon [16].

### Management

The estimated number of working hours per week varied from 1.7 to 9.3 per 1000 weaners. Farmer 8 considered presence in the shed to be crucial: *“If you want a successful weaner production, you need to spend sufficient hours in the shed*”. Despite having old buildings, this farmer had very good results in the weaner unit. In general, newer housing is expected to facilitate good practices (such as sectioning and hygiene), enabling fewer working hours without compromising results. However, “*what is crucial in the weaner production is to LOOK at the weaners, rather than at the calendar, to decide when it is time to sort them or change their feed*” (Farm 8). All farmers sorted the weaners to some extent, though the strategy varied. In general, farmers sorted by size, while a small number also sorted by sex. Sorting by sex enables differentiated feeding, which may increase the meat percentage and feed conversion, and may have some effect on the prevalence of tail biting [23].

In three of the farms (Farms 2, 5 and 8), the smallest weaners (<6 kg) were placed in a pen with fewer pen-mates and given a high quality feed mixture, and milk formula or sugar water was eventually added to increase the appetite. Under these conditions, initially small weaners had a higher growth rate and were therefore able to catch up with the larger weaners during the weaner period. The majority of farmers selling 30 kg pigs found it important to deliver a high quality product, since: “*Those 30* *kg pigs entering that truck is my public image*” (Farm 3).

### Internal biosecurity and pen hygiene between batches

All participating farms claimed to have an all-in-all-out production system. However, the extent to which this practice was managed differed between farms. Where sectioning was not practiced 100% efficiently, the design of the housing was typically regarded as a limiting factor. For example, a shed previously used for cattle had been transformed into a pig shed (Farm 11) and in another farm, productivity exceeded the intended housing capacity (Farm 5). Farm 5 did not observe clinical diarrhea in the majority of weaners kept under strict sectioned conditions (80%). However, due to the inexpedient construction of the housing, 20% of the weaners were kept in a section with continuous production where diarrhea was observed and group treatment applied regularly. Sectioning [8] and improvement of housing facilities [24] has previously been found to influence the antimicrobial treatment frequency in pig farms. Additionally, a recent study by Laanen et al. [9] demonstrated that a high level of internal biosecurity (in terms of disease management) had a protective effect on the use of prophylactic group treatments, possibly due to a reduced transmission of pathogens within the farm.

In terms of hygiene between batches of pigs, it is recommended to wash, disinfect (for a minimum of 30 min), and subsequently leave pens empty for at least 2 weeks in order to reduce the transmission of *Lawsonia intracellularis* [25]. None of the participating farms were left idle for this time period, possibly due to the associated loss of income or lack of shed capacity. Nielsen et al. [8] found that the risk of antimicrobial group treatment in finisher farms increased by a factor of four, when the housing was never cleaned. Likewise, Laanen et al. [9], identified a positive correlation between cleaning and daily weight gain, possibly due to the reduction of gastrointestinal disorders.

### Feeding

Good feeding practices may contribute to a healthy gastrointestinal microbiota, preventing diarrhea. More than half of the participating farms typically experienced diarrhea at shifts in feed (Farms 2, 3, 7, 8, 9 and 11), while only two farms did not observe diarrhea as the main clinical indication for treatment (Farms 1 and 6). Both mentioned the feeding as the reason: “*Diarrhea? No I adjust the feeding*” (Farmer 6). Whenever feces softened, they would decrease the grind of the feed slightly (Farm 6), or add a lactic acid bacteria starting culture (Farm 1). Farm 1 also added lactic acid bacteria starter culture in the feed for newly-arrived weaners. This is in accordance with prior scientific studies, demonstrating how probiotic bacteria, *Bifidobacterium lactis* Bb12 and *Lactobacillus rhamnosus*, may inhibit the adhesion of *Salmonella* sp.*, Clostridium* sp. and *Eschericia coli* to the intestinal mucosa [26].

### Treatment procedures

Farmers were asked to estimate the percentage of weaners treated in each batch, resulting in estimated treatment percentages ranging between 5 and 100%. Based on veterinary directions, the farmer chose when to initiate treatment, how to treat, the duration of treatment and what dose to use. All four parameters are highly dependent on the owner setting the standards of the farm, as well as the person in charge of the daily routines. The initiation of treatment depends on the ability to detect diseased animals, as well as the willingness of the farmer to tolerate the clinical signs. As the owner of Farm 3 stated: “*When you choose to have a low use of antimicrobials, you need to accept a certain level of diarrhea among your weaners*”. This farmer rejected group treatment as “*It’s a principle!*” In his experience, if the clinical diarrhea did not affect the general condition of the weaners they would recover without treatment. However, an “injection-only-strategy” has a considerable influence on the workload and subsequent labor costs, and can therefore be followed only by farms with the available resources. However, it can be argued that a high number of injections may stress the pigs and subsequently reduce welfare.

According to farmer No 3, the ability to detect diseased pigs and to initiate treatment at the optimal time is highly dependent on the person in charge of the daily routines. “*Some have the talent, while others will never learn*” (Farm 3). Hence, a person, which by the farmer may be characterized as talented, may use more antimicrobials in striving towards higher levels of health, welfare and productivity among the pigs. On the other hand, initiating early treatment may reduce transmission of disease and thus decrease the total amount of antimicrobials needed. However, some of the specialized pig veterinarians contacted during the initial study confirmed that farms with the highest level of health and productivity were not necessarily those using the lowest amount of antimicrobials.

More than half the participating farms administered antimicrobials in smaller units than on the section level (Farms 1, 3, 4, 5, 6, 7). One farm had two water pipes per section, enabling treatment of half a section at a time (Farm 7), while another had installed a medicine dispenser on each pen (Farm 4). Group treatment, where antimicrobials are administered through feed or water to a group of pigs, is widespread in pig production [27, 28]. Antimicrobials added to water are administered through a dispenser coupled to the water pipe. Hence, the configuration of the water pipes and/or dispenser types may have an impact on the number of treated animals at the farm, which may lead to a higher consumption of antimicrobials the larger unit each dispenser relates to.

Results from this study revealed some incongruence between recorded data and reality. In Farm 1 only 5% of the pigs received treatment. Despite this low treatment frequency, the apparent antimicrobial use as stated in VetStat was higher than expected (4.16 and 7.19), compared to farms treating 20% (6.03, Farm 5) and 50% (3.9 and 5.7, Farm 2) of their pigs. Pigs in Farm 1 stayed in the same section from a weight of 7 kg until slaughter. Antimicrobials were mainly being prescribed for weaners, but essentially administered after the pig had exceeded 30 kg of weight. This account was confirmed by the amount of antimicrobials being prescribed for finishers, which was close to zero. The treatment of a pig of 45 kg accounts for one ADD/100 finishers/day, but three ADD/100 weaners/day. This is based on the calculation of ADD, using 15 kg as a measure for a standard weaner and 50 kg as a measure for a standard finisher. As the antimicrobials were prescribed for weaners but used for finishers, the actual amount of ADDs for weaners at Farm 1 is expected to be markedly lower than stated in Table 1. Observations like this, elucidates the incongruence existing between VetStat data and use of antimicrobials in real life. Hence, in farms housing more age groups, it is essential for the veterinarian to be observant towards which age group of pigs actually is treated. When evaluating the antimicrobial use as ADD/100 pig/day, it is important to keep in mind that it is a statistical measure created to enable comparison of the relative consumption between farms, and is not necessarily a measure of the actual amount of antimicrobials used at the farm [19].

The impact on antimicrobial use of some of the factors discussed above, are supported by a currently unpublished study performed by Dupont et al., which investigates key factors which are related to a reduced use of antimicrobials. Dupont et al. found vaccination strategy and treatment method (smaller dosage, fewer group treatments, shorter treatment duration and changes in antimicrobial product) to be pointed out by farmers and veterinarians as the most important reasons for a decreased use of antimicrobials. Additionally, changes in feeding and increased compliance towards all-in-all-out procedures were mentioned.

## Conclusions

According to register data, participating farms were alike; low use of antimicrobials, mortality and high daily growth. However, on-farm interviews elucidated more heterogeneity among farmers than expected. Most of the farmers had a specific point of focus which they considered to be crucial for their good results. Points of focus mentioned by the farmers included feeding, treatment strategy, refurbishment of facilities and presence in the shed. These results indicate the importance of studies going beyond register data. Qualitative study techniques are needed striving towards a better understanding of the actions taken behind data. Further studies on the effect of farmer types are recommended.

## Endnotes

^a^Averages of daily weight gain and mortality are calculated as national averages of efficiency-control data from a representative sample of farms. The parameters are calculated as annual averages based on the number of inserted pigs.

^b^SPF, or Specific Pathogen Free farms, is a trademark of pig farms which ensures a certain level of external biosecurity through the restriction of entering visitors, equipment, feed and pigs. Hence, entering pigs need to come from another SPF farm with identical or higher health status [29]. Farms can be free from all (SPFX-) or some of the following: Porcine Reproductive- and Respiratory Syndrome (PRRS), *Actinobacillus pleuropneumoniae* (Ap), *Mycoplasma hyopneumoniae* (Myc), haemolytic *Serpulina hyodysenteriae* (Dys), toxin-producing *Pasteurella multocida* (Nys)*, Haematopinus suis* and *Sarcoptes Scabiei* var. suis. If diagnosed with a disease, the abbreviation appertaining the pathogen is added as e.g. +Ap2 (presence of *Actinobacillus pleuropneumoniae*, serotype 2).
